# The Effect of Maternal Stress Activation on the Offspring during Lactation in Light of Vasopressin

**DOI:** 10.1155/2014/265394

**Published:** 2014-01-14

**Authors:** Anna Fodor, Dóra Zelena

**Affiliations:** ^1^Institute of Experimental Medicine, Hungarian Academy of Sciences, Szigony utca 43, 1083 Budapest, Hungary; ^2^János Szentágothai School of Neurosciences, Semmelweis University, Üllői utca 26, 1085 Budapest, Hungary

## Abstract

Although it is obvious that preconceptional effects as well as stressors during pregnancy profoundly influence the progeny, the lactation period seems to be at least as important. Here we summarize how maternal stressors during the lactation period affect the offspring. As vasopressin is one of the crucial components both for stress adaptation and social behavior, special emphasis was given to this neuropeptide. We can conclude that stressing the mother does not have the same acute effect on the hypothalamo-pituitary-adrenocortical axis (as the main target of stress adaptation) of the pups as stressing the pups, but later endocrine and behavioral consequences can be similar. Vasopressin plays a role in acute and later consequences of perinatal stressor applied either to the mother or to the offspring, thereby contributing to transmitting the mothers' stress to the progeny. This mother-infant interaction does not necessarily mean a direct transmission of molecules, but rather is the result of programming the brain development through changes in maternal behavior. Thus, there is a time lag between maternal stress and stress-related changes in the offspring. The interactions are bidirectional as not only stress in the dam but also stress in the progeny has an effect on nursing.

## 1. Introduction

A lot of evidence suggests the importance of early lifetime events in altering both behavioral and several biological parameters underlying pathological changes in adulthood. Mothers form a functional unit with their offspring. It is obvious that during pregnancy the connection is so close that any effect, like stressors, influences both the mother and the pups [1, 2]. Parental lifetime exposures to environmental challenges (preconception stressors) are also associated with altered hypothalamic-pituitary-adrenocortical (HPA) activation and increased offspring neuropsychiatric disease risk by germ cell epigenetic reprogramming [3, 4]. However, after birth the offspring start a separate life. Thus, during the lactation period the strong connection begins to sway; therefore stress in mothers does not map absolutely one to one in the descendant. On the other hand, young mammals are dependent on their mothers for nourishment and tactile stimuli (early social contact), which can be affected by maternal stressors [5]. In our paper we will focus on the mother-infant relationship during the lactation period and try to clarify how maternal stressors affect the offspring. Because of the pathological consequences most of the studies focus on long-term effects, but these are secondary to acute effects. Therefore, we try to concentrate mainly on this later topic.

Adaptation to stress is a basic phenomenon in life and the HPA axis forms the basis of this adaptation [6]. The hypothalamic component of the axis consists of corticotropin-releasing hormone (CRH) and vasopressin (AVP), which act in a synergistic manner to stimulate the release of adrenocorticotropin (ACTH) from the pituitary. ACTH reaches the adrenal gland through the general circulation and stimulates the synthesis and release of glucocorticoids from the zona fasciculate (mainly corticosterone in rodents and cortisol in humans). These end-hormones are responsible for most of the effects like elevation of blood glucose level, focusing attention, dampening the immune response, but other molecules of the axis also contribute, although to a lesser extent [7, 8].

It has been recognized that, besides many other role, including water reabsorption, AVP is critical for stress-coping [9] and contributes to stress-related psychiatric disorders [10–13] and to inflammatory and autoimmune illnesses like multiple sclerosis [14]. Moreover, AVP exerts an important role in affiliative behaviors in all vertebrates, especially in social recognition/memory [15, 16] and pair bonding [17]. The female brain AVP system was suggested to be responsible for the maintained pulsatility of the HPA axis during pregnancy [18]. Moreover, AVP secretion is activated around parturition and during lactation (peak on the day before parturition) [19]. In that context in a previous study we confirmed the involvement of AVP in the behavior of lactating mothers, especially in licking-grooming their pups [20]. Based on human studies, copeptin, a stable by-product of AVP synthesis, is a highly sensitive marker of perinatal stress [21].

Taken together AVP may participate in regulating acute and long-term effects of stress both in mothers and in pups and is a fundamental component of social attachment. We used mainly the spontaneous AVP-mutant Brattleboro strain as a model organism to demonstrate the role of AVP in these processes [26, 27], but relevant literature with other tools (e.g., AVP antagonists) was also cited.

## 2. Stress Reactivity in Lactating Mothers

### 2.1. In General

During lactation female rats undergo numerous changes for better interaction with the offspring [35, 38]. Among them, alterations in basal HPA functions are especially important [39]. However, hypothalamic changes in CRH mRNA level are controversial (Table 1), but there is an increase in AVP-CRH colocalization in the paraventricular nucleus of the hypothalamus (PVN) [28]. Moreover, during lactation the ACTH response to CRH is blunted not only in rodents [40], but also in humans [41], whereas AVP triggers an increased ACTH release [42]. This suggests a shift in sensitivity of the pituitary corticotroph cells from CRH to AVP. This could be one of the most important components of the adaptation leading to—among others—reduced anxiety and attenuated stress responsiveness in mothers, which are necessary to normal postnatal development of the offspring [43]. The resting blood ACTH levels in mothers are equal [30] or even lower compared to virgins [29], with higher corticosterone levels. However, there is a flattened diurnal corticosterone rhythm in lactating dams [44]. The inverse relationship between ACTH and corticosterone suggests the involvement of other stress-related molecules (AVP, prolactin, catecholamines, and oxytocin) in the regulation [29].

**Figure 1:**
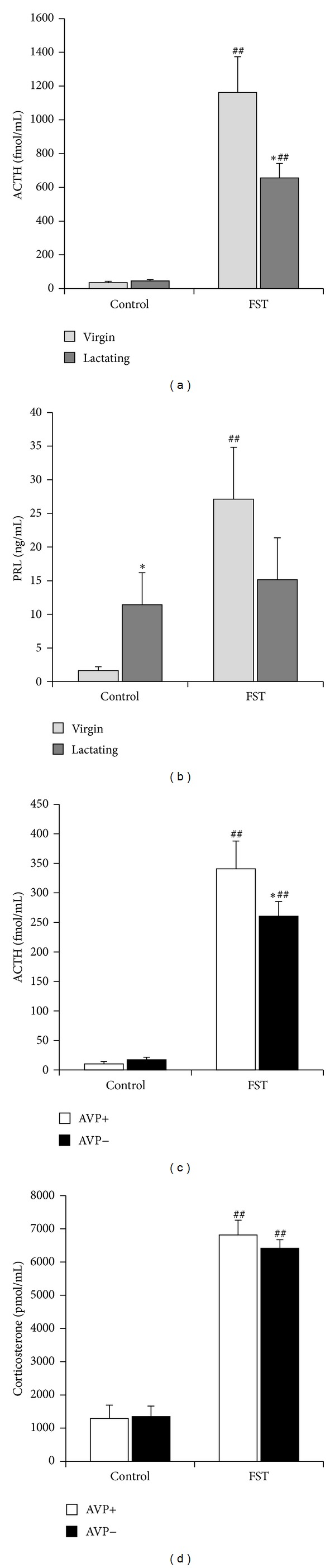
Serum hormone levels at rest (control) or at the end of forced swim stress (FST; description of the stressor: [20]). ((a), (b)) Normal AVP containing virgin and lactating (suckling 7–11-day-old pups) females were compared after 5 min FST. *N* = 8–10 ACTH secretion was smaller in dams, while resting prolactin levels were already elevated and there was no further increase in dams. The corticosterone levels were not elevated in FST animals at this time point. ((c), (d)) Normal dams were compared to AVP-deficient Brattleboro mothers after 15 min FST. *N* = 9–11 The stressor-induced ACTH elevation was smaller in AVP− animals, but there was no difference between the genotypes in corticosterone rises. *P < 0.05 versus virgin or AVP+; ^##^ *P* < 0.01 versus nonstressed control.

Indeed, observations in the lactating rat have shown an endocrine hyporesponsiveness to physical and psychological stressors, including attenuated secretion not only of ACTH (Figure 1(a)) and corticosterone [45], but also of prolactin (Figure 1(b)), catecholamines, and oxytocin [7].

Prolactin is a peptide hormone primarily produced by the anterior pituitary gland which is important in maternal milk production (during pregnancy it promotes lobuloalveolar development and after birth it stimulates lactogenesis in the mammary gland). Moreover, it can directly ensure nutrition and display of maternal behavior. The elevated prolactin level may be an important factor in the blunted HPA response that occurs during lactation: experimentally induced hyperprolactinaemia attenuates the neuroendocrine stress responses [46, 47]. Torner et al. supposed a receptor-mediated attenuation of the responsiveness of the HPA by prolactin: the stress-induced increase of ACTH secretion was decreased after chronic intracerebroventricular infusion of prolactin in virgin female rats and, in contrast, was further elevated by antisense targeting of the brain prolactin receptors [48]. On the other hand, stressor exposure induces no further prolactin elevation in lactating dams [34] (Figure 1(b)).

Catecholaminergic system is another important limb of stress adaptation [49]. Reduced noradrenergic input activity in the central nervous system (more specifically in the PVN) is also involved in reduced stress responses during lactation [47]. Regarding the peripheral catecholamine release from the adrenal medulla, Higuchi et al. showed significantly smaller increases in plasma concentrations of adrenaline and noradrenaline induced by stress (immobilization) in lactating than in nonlactating rats [34]. Lactation per se is an effective stimulator of adrenaline and noradrenaline release [50].

In the PVN both the oxytocin mRNA level and the number of oxytocin immunoreactive cells are higher in lactating dams compared to ovariectomized or late pregnant animals [33]. Moreover, the plasma oxytocin levels are also enhanced in breast-feeding mothers [51]. This greater overall oxytocin level may also contribute to alteration in stress reactivity of lactating subject, although acute stressors are unable to induce significant increase or decrease in the oxytocin plasma levels [52].

### 2.2. In Light of Vasopressin

As pregnancy-lactation is a chronic load to the organism [39], we might assume that the regulation of the HPA axis will be similar in dams than during chronic stress situations. Because there is a shift in colocalization from predominant CRH production to AVP synthesis in PVN, therefore some authors suggested AVP as the main hypothalamic regulator of the HPA axis during chronic stress [53, 54]. Our recent studies in Brattleboro dams support the involvement of AVP in the maintenance of enhanced resting HPA activity [30]. Namely, the chronic stressor-like elevated adrenal gland weight, increased mRNA levels of CRH in the PVN, and resting plasma corticosterone levels were missing in AVP-deficient mothers. Moreover, when we examined the acute stress reactivity, we found a reduced ACTH secretion in AVP-deficient dams compared to respective control mothers (Figure 1(c)). But this diminution was comparable to the effect of AVP-deficiency in virgins and did not cover the corticosterone levels (Figure 1(d)).

Taken together, during lactation suckling the young provides a neural stimulus that dampens the HPA axis circadian rhythm and reduces stress responses [47]. All HPA axis changes are reversed—at least partially—14 days after experimental weaning [44]. Thus, the presence of the pups has an important influence on regulation of the HPA axis. The neural stimulus of suckling may lead to increased activity of brain systems with inhibitory effects on the HPA axis (such as the prolactin and oxytocin systems) and to reduced activity of excitatory pathways (noradrenaline, CRH, and opioids) [55]. During lactation, AVP might have a special role in the maintenance of basal HPA activity, but the primary function of AVP in acute HPA axis regulation may be similar to that in males and virgins, namely restricted to ACTH secretion.

## 3. Separation in the Offspring

As most of the stressors are associated with more or less maternal separation (MS) and/or reduced maternal care of the offspring, we try to examine independently the separation-induced changes and the effect of other stressors.

Many studies have demonstrated—mainly in the rat—that a single or repeated separation of the pups from the mother leads to acute as well as long-term effects on endocrine system and behavior [56].

### 3.1. Acute Effects

#### 3.1.1. In General

**Figure 2:**
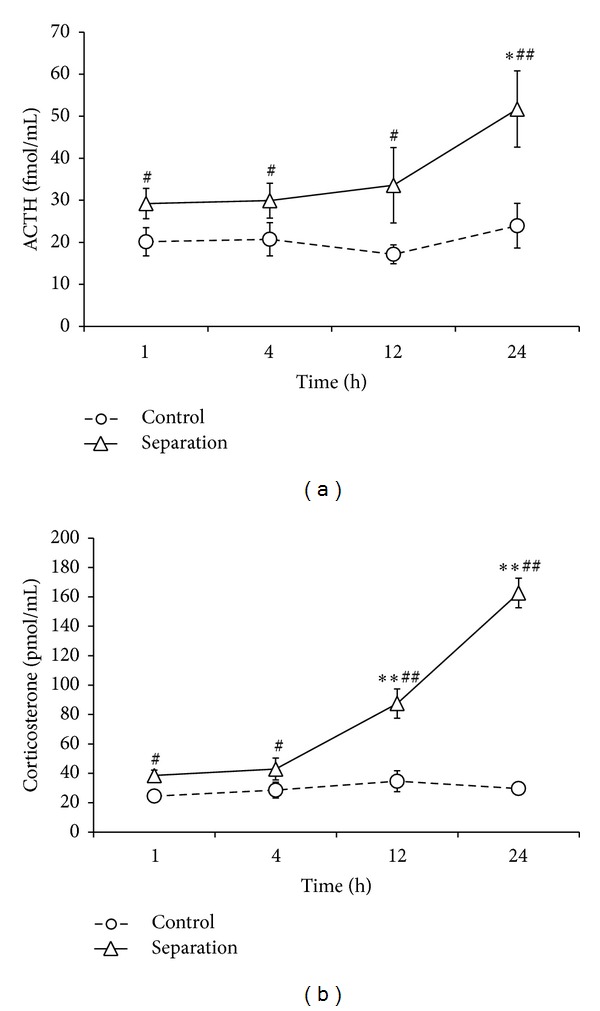
Serum level in 9-10-day-old pups (control Brattleboro rats) after different separation time from their mothers. *N* = 9–15 Single 1 h maternal separation was already able to induce serum ACTH and corticosterone rises, but prolonged separation had stronger effects (for methods see [22]). *P < 0.05, **P < 0.01 versus 1 h separation; ^#^ *P* < 0.05, ^##^ *P* < 0.01 versus nonstressed control.

The first two weeks of life called stress hyporesponsive period (SHRP) reflected the reduced HPA axis activation to stressors compared to older animals [57]. The amplitude of both ACTH and corticosterone responses increases as the function of age [22, 58], but there is no sympathetic activation to MS measured by plasma adrenaline and noradrenaline levels at preweaning [59]. Maternal components (tactile stimuli, feeding, passive contact) play a crucial role in dampening the offspring's HPA axis [60]. The longer the MS is, the stronger the HPA activation (Figure 2) [61]. When the MS pups are normally fed during separation the HPA axis is not activated [62, 63]. Around weaning, feeding seems to just partly contribute to MS-induced ACTH changes, but it is the most crucial component for corticosterone rises [64]. A similar ACTH-corticosterone discrepancy can be detected for stroking, as it reduces the MS-induced ACTH elevation but is ineffective on corticosterone changes [62]. Indeed, social factors are also very important in determining the response of the HPA system to stress not only during infancy but also in adult life [65]. The presence of familiar social partners can reduce or eliminate the glucocorticoid response to either loss of a significant social relationship or to fear inducing stimulus. There are no morning-evening differences in either the pattern or the magnitude of the ACTH or corticosterone response to MS [58].

A desensitization occurs after repeated separation [66]; however maternal care after reunions did not show similar habituation, as a possible mechanism that altered metabolism (measured by glucose and ghrelin levels) as well as glucocorticoid feedback was closed out [67]. Mineralocorticoid receptor changes seem to be the most important contributors.

It should be noted that reduced corticosterone binding globulin (CBG) in the neonate may significantly influence the interpretation of the previous results. It was demonstrated that despite lower total corticosterone concentrations the hippocampal glucocorticoid receptor occupancy/translocation was generally comparable across all ages either under basal conditions, or following stress, suggesting similar effects [68]. On the other hand, glucocorticoids may induce plasticity of other neural circuitry especially in those regulating the HPA axis [69]. These alterations may lead to a spectrum of HPA abnormalities, including aberrant HPA circadian rhythms, abnormal HPA response to stress, and basal HPA dysregulation resulting in psychopathologies.

#### 3.1.2. In Light of Vasopressin

It seems that the CRH system of neonates is not fully matured [70]. In contrast, the regulation of hypothalamic AVP gene expression matures very early [71]. Thus, AVP may be the major factor that controls ACTH release during the SHRP [60, 72, 73].

**Figure 3:**
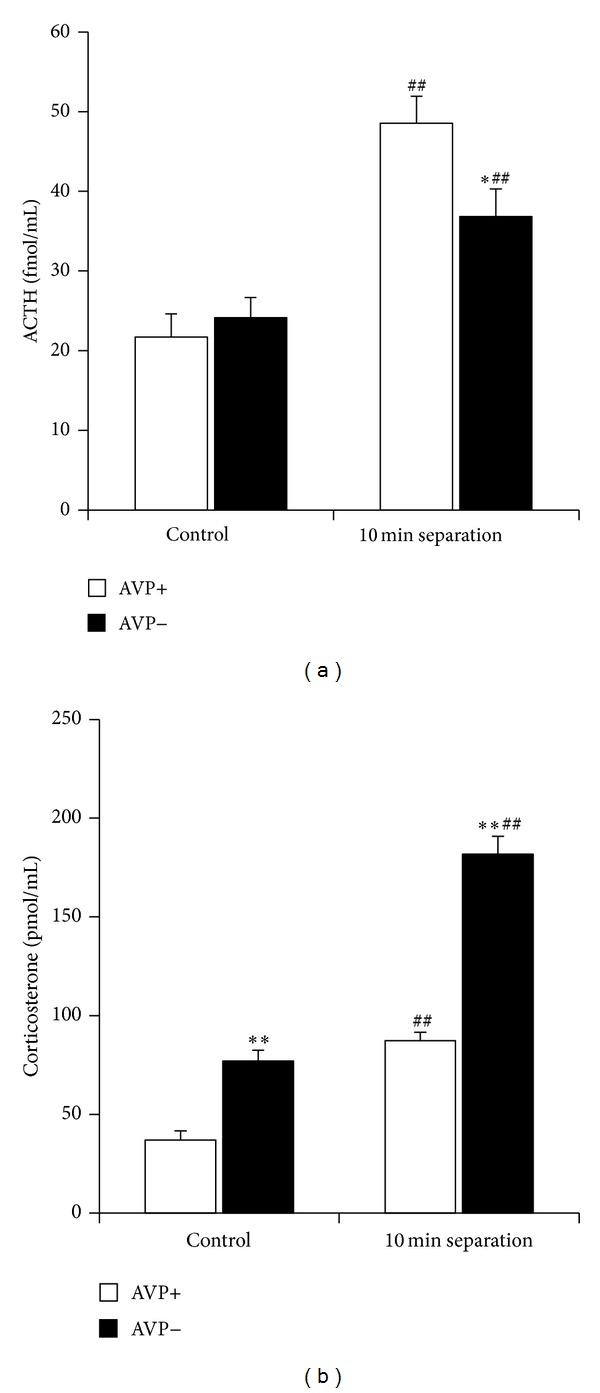
Effect of 10 min maternal separation (single housed without bedding in a 2 L glass jar) on serum hormone levels in 7-day-old control and AVP-deficient Brattleboro rat pups. *N* = 21–28 This short separation was able to increase both the ACTH (a) and corticosterone (b) levels in the offspring. However, AVP-deficient animals had smaller ACTH reaction to stress, while their corticosterone elevations were even higher than in normal animals. *P < 0.05, **P < 0.01 versus AVP+; ^##^ *P* < 0.01 versus nonstressed control.

Indeed, studies in Brattleboro rat pups showed that without AVP the ACTH elevation to 1–4–12–24 h MS is significantly reduced [22]. Already 10 min separation induces smaller ACTH activation in AVP-deficient animals (Figure 3(a)). This phenomenon was confirmed in subsequent studies using V1b receptor antagonist (the specific AVP receptor found on pituitary corticotrophs) and AVP antiserum [74]. Not only hormonal changes but also the separation-induced ultrasonic vocalization is influenced by AVP. Namely a V1b receptor antagonist was able to diminish the separation-induced distress measured by reduced ultrasound vocalization [75]. However, there is a big discrepancy between ACTH and corticosterone regulation, as stressor-induced corticosterone changes are even higher in AVP-deficient animals compared to controls (Figure 3(b)). This raises two questions: (1) what other factors contribute to the glucocorticoid secretion during the perinatal period [76]? and (2) what is the role of ACTH if not the regulation of glucocorticoid secretion [77]? Later studies were able to close out the role of enhanced adrenal gland sensitivity to ACTH or enhanced CBG levels and confirmed the involvement of beta-adrenergic regulation in the direct adrenocortical regulation [77]. A possible extra-adrenal effect of ACTH could be immune cell regulation and a life-long hormonal imprinting ([78]; for a summary see [77]).

**Figure 4:**
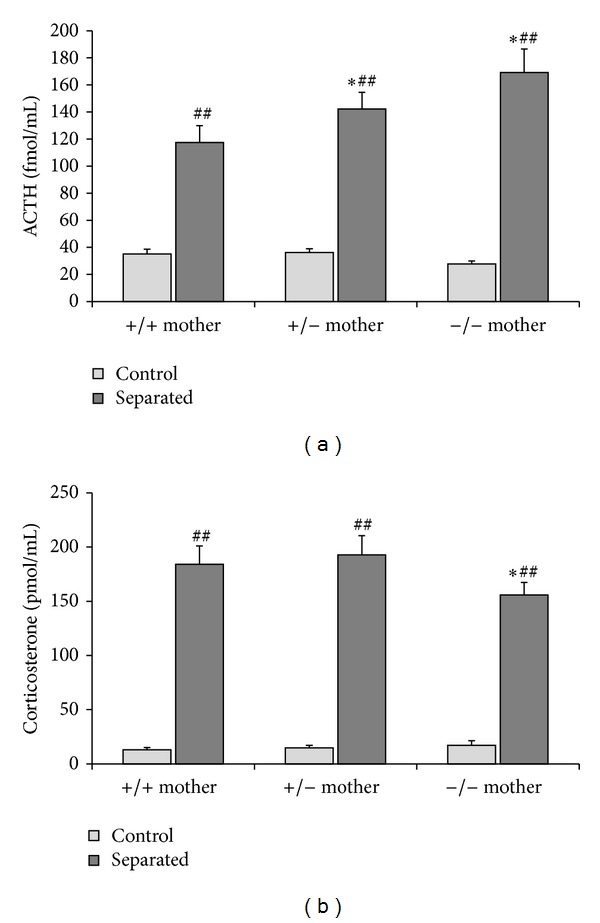
Effect of maternal genotype on acute stress reactivity of the 9-day-old pups to 24 h maternal separation (for details see [23]). *N* = 13–20 (a) Serum ACTH increase was enhanced when the one or two AVP gene was missing from the mother, while the corticosterone (b) rises were higher in the offspring of homozygous AVP-deficient mothers. *P < 0.05 versus +/+ mother; ^##^ *P* < 0.01 versus nonstressed control.

The strong connection between maternal and pup's HPA axis is further supported by the influence of maternal genotype on the offspring's stress reactivity (Figure 4). The lack of AVP in the mother resulted in an increase of 24 h MS-induced ACTH secretion in the pups, while the corticosterone rise was reduced. This could be the consequence of reduced maternal care [20] or blunted HPA activation [30] in AVP-deficient mothers. Several studies suggested a role of AVP in maternal care [79–81]. For example, the rats showing low trait anxiety represent maternal neglect underlined by reduced AVP levels in their hypothalamus [82]. In these respects, they are similar to AVP-deficient Brattleboro rats.

Interestingly, not only maternal behavior but also the development of parent-infant bonding for fathers may be related to AVP [83]. AVP in fathers increases after the birth of the child in a way analogous to the oxytocin level of the mother [84]. The paternal behaviors of marmoset fathers during the first month of the infant's life are associated with a rapid increase of AVP receptors in the prefrontal cortex of the brain [85]. Perhaps human fathers with low levels of AVP may have difficulties with parenting behaviors and may be more vulnerable to depression.

### 3.2. Long-Term Consequences

#### 3.2.1. In General

Adversity early in life elicits developmental adaptations, which are adaptive in their nature, but may later prove to be maladaptive or disadvantageous [86]. Countless animal studies showed that exposure to early-life stressors—in the form of various periods of MS, administration of exogenous corticosterone, and variable feeding conditions—modulates the regulation of defensive responses (e.g., behavioral fearfulness/anxiety and endocrine stress reactivity) in adulthood, a research field pioneered by the work of Seymour Levine and Victor Denenberg [87]. In humans, the high initial damage load (HIDL) hypothesis was formulated based upon the observations that early-life events may affect survival in later adult life [88]. The special importance of these observations is that even small progress in optimizing the early developmental process can potentially result in remarkable prevention of many diseases later in life. Although most of the authors focus on these late consequences, we think that for prevention purposes studying acute changes are equally important.

Maternal separation (MS) in rats is a well-established animal model for early life stress. Alterations in maternal care, milk composition, and pup consumption during the early postnatal period may contribute to long-term changes induced by MS [89]. Therefore, a distinction should be made between short-term separation (SMS; brief MS or early handling, EH, repeated separations for 15 min) and long-term maternal separation (LMS; MS during the stress hyporesponsive period for 180–360 min each day) or extended single MS (for 24 h) [90]. The first (SMS) stimulates development possibly through enhanced maternal care, while the latter results in reduced maternal care in combination with reduced consumption [87, 91]. One of the first descriptions by Levine indicated that repeated handling (SMS) led to reduced stress-responsivity in adult animals [92]. In later studies handled animals showed a smaller increase in plasma ACTH and corticosterone levels in response to stress than nonhandled animals most probably as a consequence of higher negative-feedback sensitivity to glucocorticoids [93]. In line with this, as adults, the offspring of mothers that exhibited more licking and grooming of pups during the first 10 days of life showed reduced plasma ACTH and corticosterone responses to acute stress, increased hippocampal glucocorticoid receptor mRNA expression, enhanced glucocorticoid feedback sensitivity, and decreased levels of hypothalamic CRH mRNA [94]. Their stress-related behavioral profile was also more favorable [95].

There are many reports and reviews stating that LMS leads to effects opposite to those of SMS [87]. Indeed, LMS increases behavioral and endocrine responses to stress [96], leads to lower body weight and higher levels of resting plasma corticosterone, accompanied by greater anxiety behavior on the elevated plus maze test in adulthood [97]. Neuropsychiatric disorders associated with early life adversity (e.g., LMS) involve neural changes reflected also in EEG [98]. In biparental zebra finches, removal of mothers alters not only the later behavior, but also the adult response of the HPA axis to an environmental stressor is increased [99]. In line with these observations, separated human beings (due to war) had higher average salivary cortisol and plasma ACTH concentrations and higher salivary cortisol reactivity to the Trier social stress test (TSST) compared to the nonseparated group [100]. Participants who had experienced separation in early childhood were more affected than children separated during infancy or school age. Bereavement stress during the first postnatal year increased the risk of offspring suicide attempt and during the second postnatal year increased the risk of autism spectrum disorder [101]. Exposure to parental incarceration in childhood is also associated with health problems (e.g., depression, anxiety, and posttraumatic stress disorder) in young adulthood [102]. In rats, the LMS-induced susceptibility to stress-triggered visceral hypersensitivity is transferred even across generations and this transfer depends on maternal care [103].

An interesting aspect is the effect of MS on dams. Eklund and coworker [104] reported that—despite the expectation—SMS in pups is stressful and anxiogenic in dams measured after weaning, while LMS is not.

#### 3.2.2. In Light of Vasopressin

**Figure 5:**
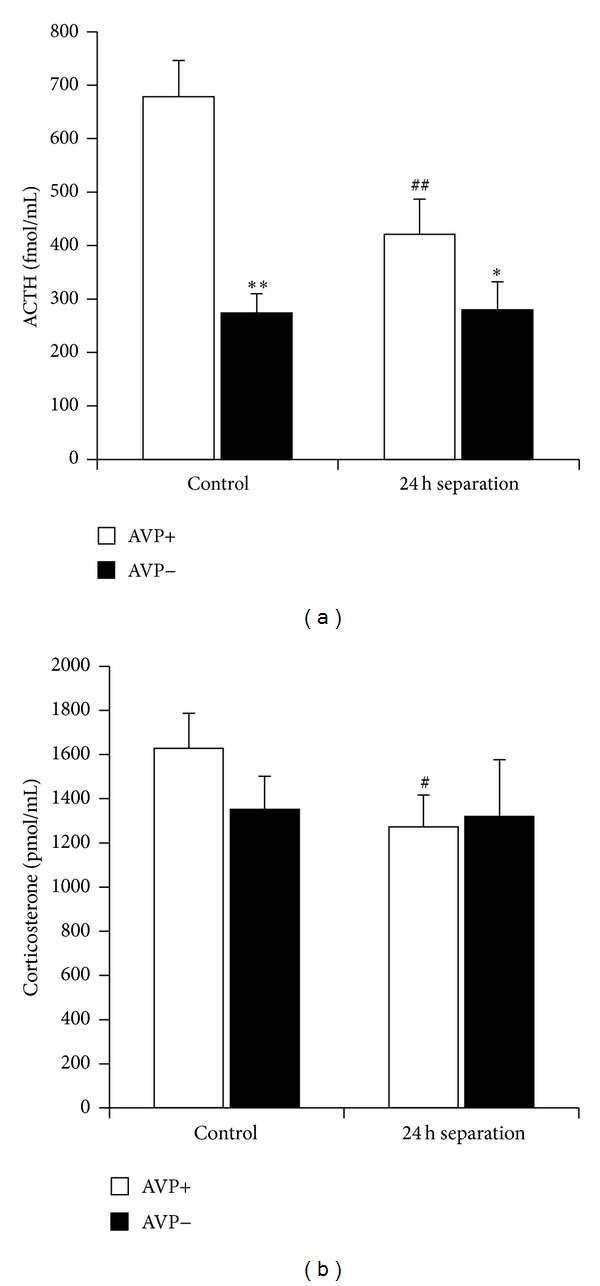
Serum stress hormone levels after 10 min openfield exposure (description of the procedure: [24]). Control and separated (single 24 h maternal separation on postnatal day 9: [22]) normal and AVP-deficient Brattleboro rats were compared at the age of 40 day. *N* = 9–15 Previous 24 h maternal separation led to reduced ACTH (a) and corticosterone (b) rises in juveniles. AVP-deficiency diminished the stressor-induced changes, without affecting the corticosterone levels. *P < 0.05, **P < 0.01 versus AVP+; ^#^ *P* < 0.05, ^##^ *P* < 0.01 versus nonseparated control.

Single 24 h MS in 9-day-old pups results in a reduced HPA activation in young adulthood (AVP+ animals on Figure 5). The ACTH activation is dampened in AVP-deficient animals (Figure 5(a)), but MS had no further effect in them, which suggests that AVP is an important factor in transmitting the MS effect on HPA axis and other neuronal plasticity.

Indeed, neonatal handling (SMS) resulted in an increased number of AVP positive neurons in the PVN accompanied by reduced social investigative interaction and increased aggressive behavior in adulthood [105]. Interestingly, LMS during SHRP also increased AVP mRNA expression in the PVN in both juvenile and adult male rats underlying enhanced adult male aggression [106]. LMS in mice leads also to enhanced AVP expression in adulthood, together with higher basal corticosterone secretion [107]. The similar changes after SMS and LMS suggest that differences between the two procedures are not always obvious, and the outcome may be profoundly influenced by experimental conditions, for example, use of proper control [87]. Another study demonstrated that exposure to LMS interferes with the developmental changes in V1a receptor binding in specific brain regions resulting in alteration in social behavior [108]. The developmental role of AVP was supported by epigenetic changes (DNA hypomethylation) on AVP gene after early life stress in mice, underpinning sustained expression and increased HPA activity [109].

**Figure 6:**
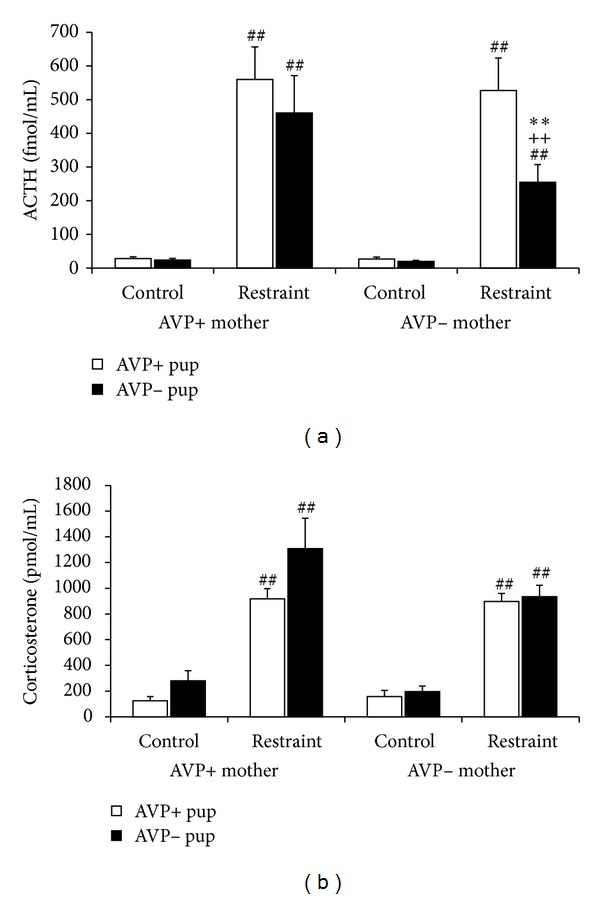
Effect of maternal genotype on the offspring's stress reactivity in adulthood. *N* = 11–16 In adults, 60 min restraint [25] significantly elevated both the ACTH (a) and corticosterone (b) levels. The ACTH elevation was smaller in the AVP-deficient offspring of AVP-deficient mothers compared both to normal progenies and to AVP-deficient offspring of normal mothers. The genotypes had no effect on corticosterone elevations. **P < 0.01 versus AVP+ offspring; ^++^ *P* < 0.01 versus AVP− offspring of AVP+ mother; ^##^ *P* < 0.01 versus nonstressed control.

Maternal AVP system is able to influence not only the acute HPA axis activation of the offspring (Figure 4), but has a profound effect on adult stress reactivity [25]. The 60 min restraint in adulthood induces significant ACTH and corticosterone rises (Figure 6), but the ACTH rise is smaller in the AVP-deficient offspring of AVP-deficient mothers compared both to normal offspring or AVP-deficient offspring of normal mothers.

Taken together MS is a widely used animal model for early life adversity. In simple terms we can say that short periods of separations (SMS) increase maternal care, which leads to better developmental skills. On the contrary, longer or prolonged separation (LMS) is harmful (for a detailed review see, e.g., [87]). Through influencing the acute HPA activation, AVP may reduce the long-term consequences.

## 4. Maternal Stress in the Offspring

Few studies have been conducted to evaluate the consequences of stressors applied to mothers on stress reactions in pups [110].

### 4.1. Acute Effects

#### 4.1.1. In General

**Figure 7:**
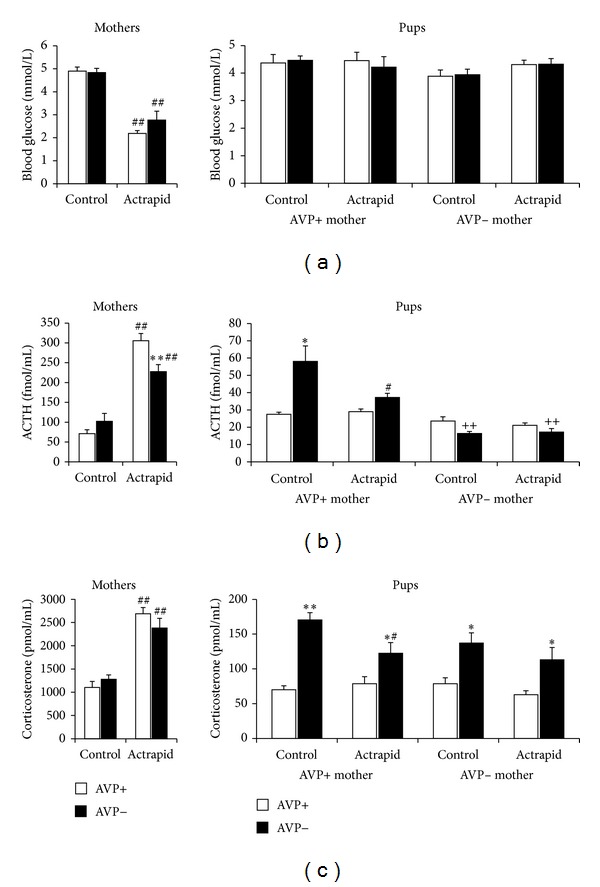
Blood glucose and stress hormone levels in normal and AVP-deficient mothers (*N* = 7-8) and in their offspring (*N* = 18–40; 7–11-day-old) after insulin (intraperitoneal injection of 3NE Actrapid) induced hypoglycemia in fasting (18 h) mothers. (a) The injection significantly diminished the blood glucose levels of mother but not their progeny, independently from the genotype. The ACTH elevations (b) were smaller in AVP-deficient dams without difference in corticosterone (c) rises. There was no increase in ACTH or corticosterone levels in the offspring. AVP-deficient offspring have higher corticosterone levels independently from the mother genotype of the stressor. *P < 0.05, **P < 0.01 versus respective AVP+ group; ^#^ *P* < 0.05, ^##^ *P* < 0.01 versus nonstressed control; ^++^ *P* < 0.01 versus AVP− control pups from AVP+ mothers.

**Figure 8:**
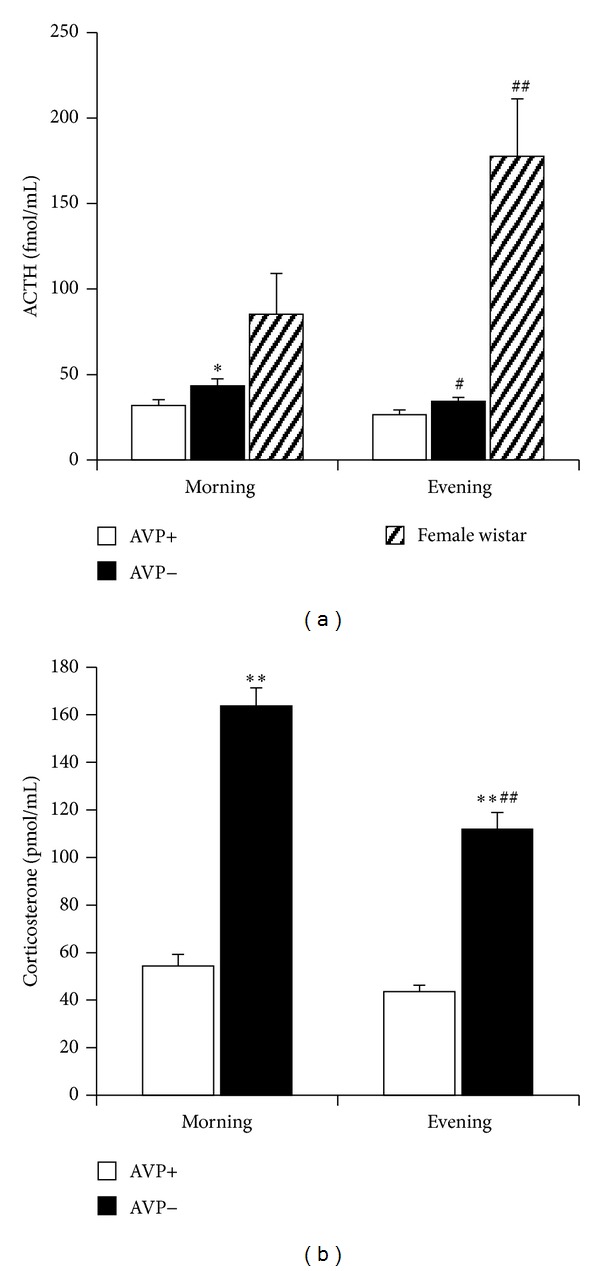
Circadian changes in 10-day-old Brattleboro rat pups compared to Wistar females. According to the textbook knowledge on rats (both in males and females) the blood ACTH and corticosterone level in adults is higher during the evening (18 h) than during the morning (8 h) hours (crossed lines). On the contrary, we found higher morning ACTH and corticosterone levels in AVP-deficient pups. *P < 0.05 **P < 0.01 versus AVP+; ^#^ *P* < 0.05, ^##^ *P* < 0.01 versus morning value.

Based on the work of Moles et al. [110, 111], we can conclude that maternal stress does not stimulate acutely at all costs the HPA axis of the offspring. Despite the repeated absence of the mother from the nest for a short period (15 min, postnatal day 2–14; mothers were stressed during this time), the otherwise unchanged environment seems to be secure enough to maintain basal activity in the offspring. Our results show similar dissociation (Figure 7). Namely, acute hypoglycemic stressor in mothers is a clear activator of the HPA axis; however, it has no effect on the offspring's ACTH and corticosterone levels. Another sign for a shift between maternal and pup's HPA axis changes is suggested by their opposite diurnal rhythm (Figure 8). Some authors found no rhythmical changes in corticosterone levels until the age of 3 weeks [112]. This could be the consequence of different nursing patterns of the different stains as well, as it is clear that the circadian changes of the offspring are strongly influenced by the mother, especially by nursing [113].

On the other hand, MS can overcome the SHRP and sensitizes the HPA axis of offspring to subsequent stressors [62, 73]. Nevertheless, 1–3 days after reunion with the mother following 24 h MS at postnatal day 3, the HPA axis activity was not hyperactive, but rather dampened, demonstrated by lower CRH mRNA and plasma ACTH and corticosterone levels [114]. Similarly, in mice, SMS for the first 14 days of life results in reduced HPA activation, although this effect was equal if the daily 15 min stressor was applied directly to pups or to the mothers [111]. Single MS does not influence the length of SHRP [114].

#### 4.1.2. In Light of Vasopressin

Insulin induced similar hypoglycemia in fasting normal and AVP-deficient mothers with smaller ACTH reaction in AVP-deficient dams (Figure 7, first columns). These changes were similar to the effect of other stressors, for example, forced swim (Figures 1(c) and 1(d)) with a dissociation between ACTH and corticosterone elevations (for a review see [76, 77]). As hypoglycemia in the mother does not result in separation from the pups; therefore the HPA axis changes can be transmitted to the offspring independently from separation. Actrapid was not transferred through the milk as no sign of hypoglycemia could be detected in the offspring (Figure 7(a)). There is no alteration in ACTH or corticosterone levels in the offspring of stressed mothers compared to nonstressed ones (Figures 7(b) and 7(c)). AVP-deficient offspring had higher corticosterone levels independently from the genotype or stress state of the mother, suggesting another mechanism (chronic load as a result of disturbed water regulatory homeostasis).

AVP is an important regulator of the circadian rhythm [115]. AVP-deficiency resulted in higher resting levels and a more expressed morning-evening difference in 10-day-old pups (Figure 8). This raises the possibility that—to some extent—the AVP system can be responsible for the reduced circadian pattern during early perinatal ages. Indeed, AVP in the suprachiasmatic nucleus (main regulator of circadian changes) shows a free-running rhythm during the nursing period [116]. In its absence in AVP-deficient pups other, more mature regulatory mechanisms may come to the fore.

### 4.2. Later Consequences

#### 4.2.1. In General

Maternal stress—despite the absence of acute HPA axis elevation in the offspring—is able to induce long-term behavioral consequences (e.g., anxiety) in offspring similar to the direct stress of the pups [110]. Thus, we can assume that in the long run maternal stressors can influence the maternal care and the composition of the milk, thereby affecting the neurobiological development of the offspring.

A widely used model of stressor induced changes is the administration of glucocorticoids. Low doses of corticosterone in the maternal drinking water, which may reflect a form of mild environmental stimulation like SMS, enhanced the offspring's ability to cope with different situations, showing improved learning capabilities and reduced fearfulness in anxiogenic situations with a persistent hyporeactivity of the HPA axis, leading to a resistance to ischemic neuronal damage [117, 118]. On the other hand, elevated doses, comparable to those elicited by strong stressors (e.g., LMS), caused developmental disruption. These observations are in accordance with the eustress-distress theory of Hans Selye and suggest that a certain level of HPA axis activation is beneficial [119].

Indeed, exercise during pregnancy and lactation prevented maternal obesity-induced elevation in corticosterone in rat offspring [120]. However, a mild form of stressor, the social instability during pregnancy and lactation, was unable to influence the resting plasma cortisol level or adrenal thyrosine hydroxylase (catecholamine synthesizing enzyme) activity in wild cavy [121]. On the contrary, the offspring exposed to maternal depression during early childhood evidenced high and increasing cortisol levels in response to a laboratory stressor [122].

An interesting finding of Levine and coworker was that shock during SHRP has similar effects on learning abilities as early handling (equal to SMS) [123]. However, they measured only lateral avoidance, but the relationship between early life experiences and adulthood learning and memory performance is multifaceted and decidedly task-dependent [124].

Changes in dietary components may induce also life-long alterations during critical windows of brain development. For example, low-folate supply during early life may leave an epigenetic mark that can predispose the offspring to further dietary insults [125]. Food and water deprivation in mother led to changes in the immune system of the offspring [78]. This effect was similar in case of direct (prenatal, in the mother) and indirect (postnatal, transmitted by milk) stress treatment, which calls attention to the danger of stress during this latter period.

Despite the hormonal alterations during lactation, tobacco smoke exposure through breast milk induced no change in resting ACTH and corticosterone level of the progeny and only programmed the adrenal medullary function at adulthood [126].

Another, clinically relevant model of early life stress is the limited access of nesting material (postnatal day 2–9) [127]. This induces chronic stress-like changes in the dam's HPA axis; however, there is no alteration in the AVP mRNA in the PVN. In adulthood—similarly to strong perinatal stressors, for example, LMS—the offspring of stressed dams showed corticosterone hypersecretion to a novel stressor and preference for “comfort food” [128].

#### 4.2.2. In Light of Vasopressin

An indirect data for the role of AVP is the effect of AVP-deficiency in Brattleboro mother on adult HPA activity (Figure 6). A similar effect can be found in the high and low anxiety mice strain [82]. Low anxiety animals have less AVP in their hypothalamus and display less maternal care. Among the offspring of low and high anxiety dams there was no difference in MS-induced ultrasound vocalization (as a sing of acute distress), but in adulthood, less maternal care was accompanied by enhanced anxiety.

Another indirect data is that mild maternal hyperthyroidism leads to anxiogenic phenotype of the progeny in their adulthood through changes in the stress regulating AVP system [129].

## 5. Conclusions

General remarks.

1. The resting HPA axis activity of lactating dams is similar to chronically stressed rats (Table 1 elevated AVP and glucocorticoids compared to virgins).
2. Further HPA activation to acute stressors is dampened in lactating dams (Figures 1 and 7).
3. The offspring show reduced HPA activation to stressors (SHRP) most probably as a consequence of maternal factors (Figure 2).
4. Maternal separation (MS) acutely activates the HPA axis of the offspring in a time-dependent fashion (Figure 2) and sensitizes it to subsequent stressors.
5. Long-term consequences of SMS (similarly to low dose corticosterone administration) are rather beneficial, while LMS (similarly to high dose corticosterone) has the opposite effect (note: methodological differences, proper control) (Figure 5).
6. Stressing the mother does not have the same acute effect on the HPA axis of the pups as stressing the pups (Figure 7), but later endocrine and behavioral consequences can be similar. Thus, it is better to speak about a shift in the HPA axis responsibility between mothers and pups and not about an absolute incoherence.

The role of AVP.

1. In dams AVP participates in the maintenance of basal HPA activity, but its role in acute HPA axis regulation is restricted to regulation of ACTH secretion (Figure 1).
2. During the perinatal period AVP is the main regulator of stressor-induced ACTH secretion in the pups (Figure 3).
3. Acute (Figure 4) as well as late consequences of perinatal stress in offspring can be influenced by the AVP system of the mother. It should be the consequences of maternal factors (milk composition, maternal care), as the lack of AVP in the dam influences the adult HPA axis reactivity even without extra perinatal stimuli (Figure 6).

Taken together, AVP plays a role in acute and later consequences (among other changes in HPA axis and stress-related psychopathology) of perinatal stressor both applied to the mother or to the offspring, thereby contributes to transmitting the mothers' stress to the progeny. This mother-infant interaction does not necessarily mean a direct transmission of molecules, but rather is the result of programming the brain development through changes in maternal behavior. The interactions are bidirectional as not only stress in the dam but also stress in the progeny has an effect on nursing [104].

## Figures and Tables

**Table 1:** Stress-related hormonal changes in the dams during lactation.

Hormone	Changes	References
Resting levels compared to virgins		
CRH	Lower mRNA levels in PVN	[28, 29]
	Higher mRNA	[30, 31]
AVP	Increase mRNA in PVN	[28, 29, 31]
ACTH	Normal	[30]
	Reduced	[29]
Glucocorticoids	Enhanced	[29, 30]
Prolactin	Elevated	[29]
Catecholamines (NA, A)	No change in hypothalamus	[32]
Oxytocin	Higher mRNA in PVN	[29, 33]

Changes after stressor exposure compared to virgins		
CRH	No increase	[31]
AVP	Enhanced colocalization with CRH; increase both in mRNA and protein	[29, 31]
ACTH	Diminished activation	[30]
Glucocorticoids	Diminished activation	[30]
Prolactin	Diminished activation	[34]
Catecholamines (NA, A)	Diminished activation	[34]

During pregnancy and lactation the hormonal system of the mother undergoes substantial changes. The best known are the changes in sexual steroids [35]. Growth hormone elevation also occurs [36]. However, suckling or stressors during lactation induced no changes in plasma thyrotrophin, growth hormone, or sexual hormone level [37]. A: adrenaline; ACTH: adrenocorticotropin hormone; AVP: arginine vasopressin; CRH: corticotropin-releasing hormone; mRNA: messenger ribonucleic acid; NA: noradrenaline; PVN: paraventricular nucleus of the hypothalamus.

## Abbreviations

ACTH: Adrenocorticotropin hormone
AVP: Arginine vasopressin
CBG: Corticosterone binding globulin
CRH: Corticotropin-releasing hormone
EEG: Electroencephalography
EH: Early handling
HIDL: High initial damage load
HPA: Hypothalamic-pituitary-adrenocortical
LMS: Long-term maternal separation
mRNA: Messenger ribonucleic acid
MS: Maternal separation
PVN: Paraventricular nucleus of the hypothalamus
SHRP: Stress hyporesponsive period
SMS: Short-term maternal separation
TSST: Trier social stress test.
